# Time‐resolved diode dosimetry calibration through Monte Carlo modeling for *in vivo* passive scattered proton therapy range verification

**DOI:** 10.1002/acm2.12210

**Published:** 2017-10-29

**Authors:** Allison Toltz, Michaela Hoesl, Jan Schuemann, Jan Seuntjens, Hsiao‐Ming Lu, Harald Paganetti

**Affiliations:** ^1^ Department of Physics McGill University MUHC Cedars Cancer Centre DS1.7137 Montreal QC Canada; ^2^ Computational Clinical Medicine Medical Faculty Mannheim Heidelberg University Mannheim Germany; ^3^ Department of Radiation Oncology Francis H Burr Proton Therapy Center Massachusetts General Hospital and Harvard Medical School Boston MA USA; ^4^ Medical Physics Unit McGill University MUHC Cedars Cancer Centre DS1.7137 Montreal QC Canada

**Keywords:** proton therapy, radiation dosimetry, TOPAS

## Abstract

**Purpose:**

Our group previously introduced an *in vivo* proton range verification methodology in which a silicon diode array system is used to correlate the dose rate profile per range modulation wheel cycle of the detector signal to the water‐equivalent path length (WEPL) for passively scattered proton beam delivery. The implementation of this system requires a set of calibration data to establish a beam‐specific response to WEPL fit for the selected ‘scout’ beam (a 1 cm overshoot of the predicted detector depth with a dose of 4 cGy) in water‐equivalent plastic. This necessitates a separate set of measurements for every ‘scout’ beam that may be appropriate to the clinical case. The current study demonstrates the use of Monte Carlo simulations for calibration of the time‐resolved diode dosimetry technique.

**Methods:**

Measurements for three ‘scout’ beams were compared against simulated detector response with Monte Carlo methods using the Tool for Particle Simulation (TOPAS). The ‘scout’ beams were then applied in the simulation environment to simulated water‐equivalent plastic, a CT of water‐equivalent plastic, and a patient CT data set to assess uncertainty.

**Results:**

Simulated detector response in water‐equivalent plastic was validated against measurements for ‘scout’ spread out Bragg peaks of range 10 cm, 15 cm, and 21 cm (168 MeV, 177 MeV, and 210 MeV) to within 3.4 mm for all beams, and to within 1 mm in the region where the detector is expected to lie.

**Conclusion:**

Feasibility has been shown for performing the calibration of the detector response for three ‘scout’ beams through simulation for the time‐resolved diode dosimetry technique in passive scattered proton delivery.

## 1 INTRODUCTION

Proton therapy may reduce adverse effects of radiation due to the potential for increased normal tissue sparing as compared to other modern external beam radiotherapy techniques.^1^ Variation in beam range is the result of variation in stochastic range straggling in the presence of anatomical tissue heterogeneity. Patient setup error, misalignment, and internal organ motion result in different heterogeneities in the path of the beam at time of treatment as compared to CT simulation. Range uncertainties are currently accounted for using range margins, but these margins limit the potential advantages of distal edge normal tissue sparing of proton therapy. Calculation of the position of the Bragg peak for a given heterogeneous tissue distribution is determined using a conversion of CT Hounsfield units (HU) to relative proton stopping power combined with measurements in water. This calibration is subject to the uncertainty of converting HU to water‐equivalent density, approximately 1%–2% of the proton beam range, as well as uncertainty in CT HU measurements themselves, mainly due to beam hardening and also approximately 1%–2% of the proton beam range.^2^ A range uncertainty estimate of 3.5% ± 1 mm of the proton beam range as a distal margin is commonly implemented in proton therapy treatment planning for spread out Bragg peak (SOBP) fields.^3^ Sparing healthy tissue by reducing the applied margins provides motivation to develop millimeter accuracy in range verification techniques. The use of a small volume detector array for range measurements has been proposed,^4^, ^5^ which could provide the opportunity for *in vivo* range verification and be used on‐line to adapt the treatment plan and minimize these margins for passively scattered proton delivery.

Because the detector response is beam‐specific, experimental measurements in homogeneous media have been employed to establish a calibration curve of the response of the detector to WEPL for every SOBP that may be delivered for each given clinical case.^4^, ^5^, ^6^, ^7^, ^8^, ^9^, ^10^ This process is both tedious, as it necessitates a separate set of measurements for every new ‘scout’ beam (a 1 cm overshoot of the predicted detector depth with a dose of 4 cGy), as well as inconvenient due to the time constraints for access to the clinical beamline. The aim of this work is to investigate whether the calibration response can be simulated with sufficient accuracy to eliminate the necessity of performing these physical measurements.

## 2 METHODS

### 2.A Time‐resolved diode dosimetry

**Figure 1:**
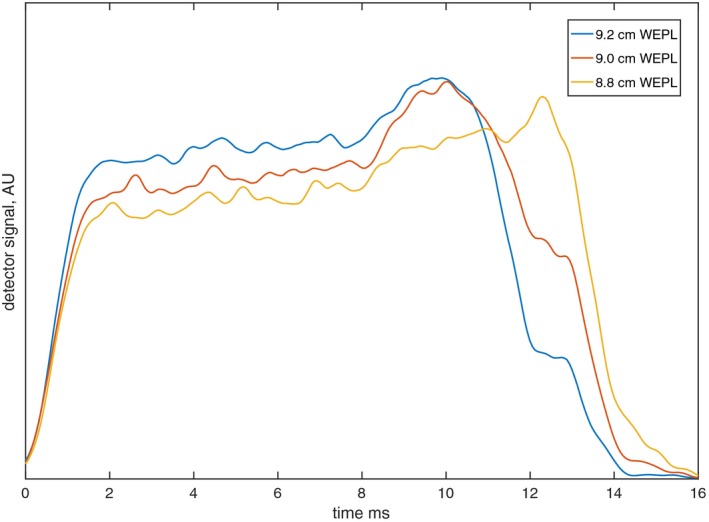
Dose rate profile per range modulator wheel cycle at 9.2 cm, 9.0 cm, and 8.8 cm WEPL for TOPAS‐simulated SOBP of 10 cm range, 9.9 cm modulation width.

The characteristic time dependence of the dose rate at a point within the SOBP can be used to determine the water‐equivalent path length (WEPL) when a deliberate overshoot of the targeted range is implemented such that the detector lies in the plateau of the SOBP.^4^ The relationship between detector signal and WEPL results from the periodic motion of the range modulation wheel (RMW) in the passively scattered delivery mechanism. A measurement of the detector signal at depth in a medium over one period of the rotation of the wheel can determine the residual range of the beam according to a set of calibration data because the dose rate profile per RMW cycle is unique to each depth (Fig. 1).

The detector signal per rotation of the RMW at a given depth (WEPL) in a homogeneous medium is specific to a given set of beam parameters: range, modulation width, and beam current modulation sequence (which varies proton fluence to achieve ± 2% flatness in the plateau region of the SOBP). These selections are made to suit the clinical application (expected depth of detector for a given treatment site) such that the ‘scout’ SOBP will overshoot the expected WEPL of the detector by 1 cm.

While in previous implementations, a set of calibration measurements in homogeneous media were required to establish a calibration, we now apply Monte Carlo simulations to achieve this calibration curve. For a system where the periodic motion and geometry of the RMW are known, the time‐varying position of the nozzle components can be implemented into a Monte Carlo framework to simulate the behavior of the proton beam as a function of the position of the wheel. A relationship can then be modeled for the scored dose over the period of rotation at a given depth of a scoring medium.

As shown previously, a statistical approach for analysis can be applied to correlate the dose rate profile per RMW cycle to WEPL.^6^ The root‐mean‐square (rms) width of each dose rate profile was computed for each detector positional depth, and a relationship between the rms width of the time the diode reads signal and WEPL was established. A fourth‐order polynomial was fitted to provide a continuous calibration curve for dose rate per RMW cycle and WEPL as per Gottschalk 2011.^6^

### 2.B Calibration measurements

Measurements were performed on Gantry 1 at the Francis H Burr Proton Therapy Center (FHBPTC) at the Massachusetts General Hospital (MGH) in Boston, Massachusetts. The passively scattered delivery mechanism was selected along with the 25 cm snout. No apertures or compensators were used. The beamline setup at FHBPTC results in an energy selection of the most penetrating Bragg peak such that the prescribed range is defined as the point of the depth dose curve at the distal 90% of the maximum dose, and the modulation width defines the distance between the points at the proximal 98% and distal 90% of the SOBP.^11^

**Figure 2:**
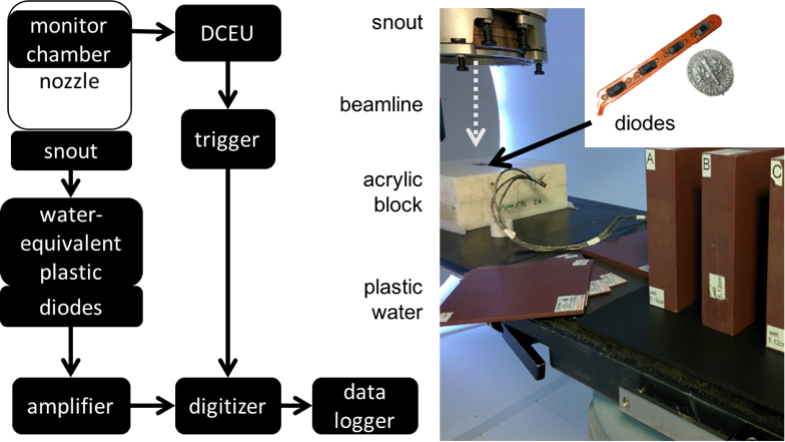
Experimental workflow (left) and experimental setup (right) for calibration measurements with diode array.

Three ‘scout’ beams were selected to provide sufficient overshoot of targets for different clinical scenarios. A 10 cm range beam with 9.9 cm modulation width, a 15 cm range beam with 14 cm modulation width, and a 21 cm beam with 18 cm modulation width were selected as representative for typical treatment fields. The ‘scout’ beams were delivered at a dose rate of 2 Gy/min, and signal for each diode over the RMW period of 100 ms was collected for 19 rotations of the RMW. Three arrays of four DFLR 1600 silicon diodes (Diodes Incorporated, Plano, TX, USA) (Fig 2), each with an active area^12^ of 0.8 mm^2^, were placed at the isocentric plane, and slabs of Gammex Solid Water^®^ (Sun Nuclear Corp., Melbourne, FL, USA) were stacked atop to create effective water‐equivalent depths. The detector response was measured at thirty depths within 10 cm of the beam range for each ‘scout’ beam investigated in minimum increments of around 2 mm (as a result of the available thicknesses of water‐equivalent plastic). The initial and final depths were selected so as to acquire data in the plateau region of the SOBP for each ‘scout’ beam. Energy dependence of the diodes was not considered as the analyzed data were the acquired signal in time, not the absolute value of the signal itself.

The diodes were connected to an in‐house amplification and digitizing system as detailed in Bentefour 2015^9^ (Fig 2), in essence comprising a preamplifier, a digitizer, and custom software for data logging and acquisition. The digitizer was triggered using data from the dose counting electronics unit (DCEU) which monitors the beam current modulation.

For a single diode, the voltage *v*_i_ was sampled at time *t*_i_ at a rate of 100,000 samples/sec. The *σ*_rms_ width of the resulting distribution was then computed, resulting in a *σ*_rms_ width value for each of 19 cycles, for each of 12 diodes, for each of 30 WEPLs, for each of three beams. Thus, over 20,000 data points were collected. The mean *σ*_rms_ width was taken over the 12 diodes and 19 cycles to determine a mean *σ*_rms_ width per WEPL, and a fourth‐order polynomial was used to fit *σ*_rms_ width as a function of WEPL.

### 2.C Simulation methods

The TOPAS platform (TOPAS MC Inc., Oakland, CA, USA), a wrapper which extends the Geant4 Simulation Toolkit, was validated by Testa et al. 2013^11^ to reproduce SOBPs at the FHBPTC at MGH with a range accuracy of ± 1 mm.^11^ It is critical that the entire beamline is accurately modeled, as the simulation results are based on the particle trajectories and interactions within the universal treatment head (which may be configured to deliver either passively scattered or scanned pencil beams). In the passively scattered configuration under investigation in this study, the most important beamline components are the RMW tracks, the first and second scatterers, and the beam current modulation selections. The recommended values of mean excitation energies from the International Commission on Radiation Units (ICRU 1993) were used to determine proton stopping powers for the materials in the treatment head.

The beam energy and nozzle settings were input into a previously validated TOPAS script MCAUTO^13^ specifying the gantry angle, range, modulation width, snout identification and extension, and number of histories (100 M) to generate the treatment head selections for the given clinical scenario. This generated a parent parameter file detailing the initial beam energy (which is then modulated by the RMW), initial beam angular spread, beam spot size, scatterers, RMW tracks and rotation, the beam current modulation sequence, and the snout selection as modeled and validated in Paganetti et al. 2004.^14^ TOPAS v2.0.p3 was used to transport particles through the entire treatment head and a water‐equivalent plastic phantom. Dose to water was scored in 1 cm × 1 cm × 1 mm voxels over the 100 ms rotation of the RMW every 0.25 ms at depths ranging from the proximal edge of the SOBP to the prescribed range in increments of 0.01 cm.

Simulations were repeated for ten different randomization seeds, since each simulation represents a single full rotation of the RMW. A fourth‐order polynomial was fitted to each set of simulated data, and the fit was used to predict the *σ*_rms_ width of the dose rate profile per RMW cycle at each of the measured depths. The standard error over the results from each seed was computed over the mean of these values for each depth.

Dose rate profiles were scored for three different absorption media. Water‐equivalent plastic was modeled using material chemical compositions and densities provided by the manufacturer, and these simulations are referred to as ‘simulated plastic water.’ A CT of water‐equivalent plastic was also used as an absorber, and finally a patient CT where the beam penetrated only soft tissues in the abdomen, with no air cavities, was used to evaluate WEPL based on dose rate profiles with depth.

## 3 RESULTS

**Figure 3:**
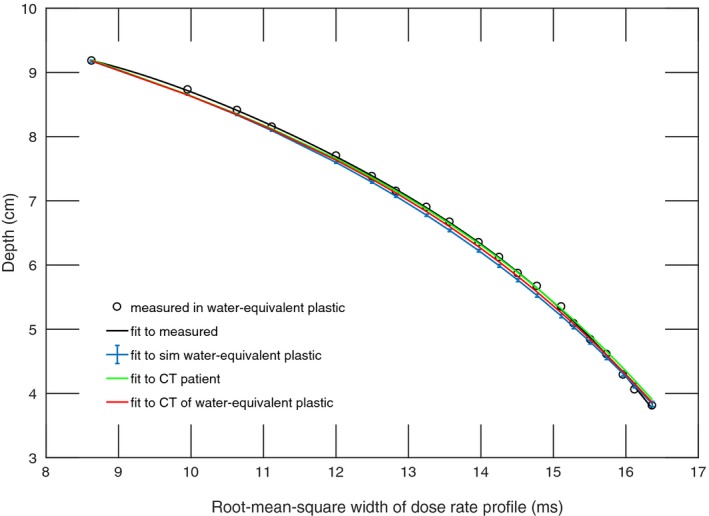
Calibration function of time‐resolved diode dosimetry system to WEPL as determined by fourth‐order polynomial fitted to measurements and TOPAS simulation of dose rate profile per range modulation wheel cycle for the SOBP of 10 cm range, 9.9 cm modulation width. Shown are the measured data, a polynomial fitted to measured data, and polynomials fitted to the simulated dose rate profiles in simulated water‐equivalent plastic, a water‐equivalent plastic CT, and a patient CT.

**Figure 4:**
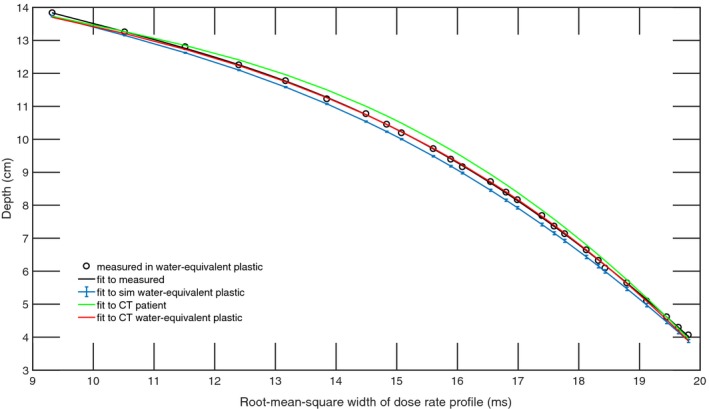
Calibration function of time‐resolved diode dosimetry system to WEPL as determined by fourth‐order polynomial fitted to measurements and TOPAS simulation of dose rate profile per range modulation wheel cycle for the SOBP of 15 cm range, 14 cm modulation width. Shown are the measured data, a polynomial fitted to measured data, and polynomials fitted to the simulated dose rate profiles in simulated water‐equivalent plastic, a water‐equivalent plastic CT, and a patient CT.

**Figure 5:**
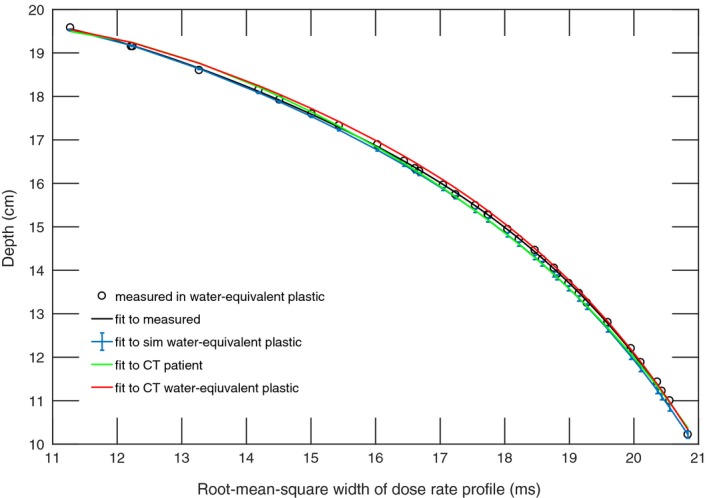
Calibration function of time‐resolved diode dosimetry system to WEPL as determined by fourth‐order polynomial fitted to measurements and TOPAS simulation of dose rate profile per range modulation wheel cycle for the SOBP of 21 cm range, 18 cm modulation width. Shown are the measured data, a polynomial fitted to measured data, and polynomials fitted to the simulated dose rate profiles in simulated water‐equivalent plastic, a water‐equivalent plastic CT, and a patient CT.

The dose at the position of the diode was simulated for the ‘scout’ SOBPs of range 10 cm, 15 cm, and 21 cm (168 MeV, 177 MeV, and 210 MeV), and the fourth‐order polynomial fits were compared against the measured data resulting in an adjusted R^2^ of 0.999 for all three beams in water‐equivalent plastic and an adjusted R^2^ of 0.998 in the patient CT (Fig 3, 4, 5). The maximum WEPL deviation of fitted values from measurements and adjusted R^2^ between the polynomial fitted to simulated data and the polynomial fitted to measured data are shown in Table 1.

**Table 1:** WEPL deviation of simulation‐derived fitted values from measurements and adjusted R^2^ for simulation‐derived calibration functions and measurements‐derived calibration functions in each of the absorption media

Beam range	Measurements in water‐equivalent plastic			Simulated in water‐equivalent plastic				Simulated in CT of water‐equivalent plastic				Simulated in patient CT			
	WEPL deviation (mm)			R^2^	WEPL deviation (mm)			R^2^	WEPL deviation (mm)			R^2^	WEPL deviation (mm)		
	Mean	σ	Max		Mean	σ	Max		Mean	σ	Max		Mean	σ	Max
10 cm	0.0	0.2	0.5	0.999	0.7	0.6	1.5	0.999	0.3	0.5	0.9	0.998	0.1	0.6	1.4
15 cm	0.0	0.3	0.9	0.999	1.6	0.5	2.4	0.999	0.1	0.6	1.5	0.998	1.8	1.2	3.4
21 cm	0.0	0.4	1.1	0.999	0.9	0.6	1.3	0.999	0.8	0.6	1.6	0.999	0.5	0.9	1.7

Within 1 cm of the range of the “scout” SOBP, where the detector is expected to lie, the deviation between measured and simulation‐derived WEPL is within 1 mm for all beams for all absorption media.

## 4 DISCUSSION

In this work, a method for the calibration of a diode detector system response was established through simulation for three “scout” SOBP beams of anticipated clinical relevance. In lieu of acquiring measurements to calibrate detector response in a novel “scout” beam, this work has validated the accuracy of using a TOPAS simulation to establish the detector response if a new “scout” beam for which no calibration fit has been measured or simulated is required.

The time‐consuming nature of acquiring these measurements, as well as limited access to beam time at a clinical facility, motivates the use of simulations to determine the calibrated detector response. Monte Carlo simulation methods do, however, introduce several sources of uncertainty which must be quantified to determine the utility of the methodology. To understand the accuracy associated with determining WEPL from the simulated dose rate, three absorption media were investigated in our simulations and compared to measured response in water‐equivalent plastic. Several sources of uncertainty exist for each case which limit the accuracy of the simulation methodology to determine WEPL. Nonuniformity and batch variability present in the measured water‐equivalent plastic as compared with the manufacturer‐supplied specifications used for the simulated water‐equivalent plastic composition are not included. Range uncertainty of 0.2% is introduced which results from CT HU to proton stopping power conversion in Monte Carlo simulations.^3^ As the maximum WEPL deviation from measurements was of the order of 1–3 mm for all three media, we conclude that there is no significant difference in the accuracy of determining the WEPL from simulated dose rate among each of the three media.

It was noted that the mean and maximum WEPL deviation of simulation‐derived fitted values from measurements were largest for the 15 cm range beam in simulated water‐equivalent plastic and in the patient CT. However, the difference among the three beams for the mean WEPL deviation with 1*σ* uncertainty is nonsignificant for all three simulation cases. This is in agreement with results from Testa et al.^11^ where agreement between TOPAS‐simulated range and measured range of the SOBPs for the RMW configuration options for our three beams was shown to range from 1.3 mm to 3.1 mm. There are numerous sources of uncertainty associated with determining simulated proton range in both the simulated water‐equivalent plastic and in the water‐equivalent plastic CT. The material composition and density values for the water‐equivalent plastic were derived from the M.Sc. thesis of Bourque^15^ as provided by the manufacturer for research purposes only. However, assumptions of uniform density and intra batch nonvariability may not be completely accurate. Additionally, an HU variability of up to 20 HU was observed in the water‐equivalent plastic CT image. This results in a relative stopping power ratio variation of 0.02 according to the methodology in Yang et al.^16^ Both simulation cases suffer from uncertainties, and a ground truth cannot be determined with sufficient accuracy based on the known information.

The methodology for establishing a fit to determine WEPL as a function of detector response using TOPAS is contingent upon an accurate treatment head model. For the beam model used in this work, the model was validated to be within clinical tolerances at FHBPTC of MGH of + 1/−2 mm for range and ± 3 mm for modulation width.^11^ Another facility aiming to implement the simulation methods for determining the calibration response of a similar time‐resolved diode dosimeter system would first need to establish and validate a beam model in the TOPAS environment.

The simulated SOBPs were observed to exhibit a 2% variation in flatness, which is within clinical tolerance, and may deviate by up to 1% from measured SOBPs.^11^ When normalizing the data prior to performing the fitting, the selection of initial and final depth indices corresponding to minimum and maximum depths for each data set was restricted to values 2 cm from the depth of maximum dose and the proximal edge of the plateau region of the SOBP.^4^

The accuracy of determining WEPL from dose rate profiles per RMW cycle is expected to decrease in the presence of range mixing as was confirmed experimentally.^7^ Nearly all clinical scenarios will involve some level of range mixing as the “scout” beam penetrates the inhomogeneous patient. In order to use the time‐resolved diode dosimetry system to accurately evaluate the WEPL to the dosimeter, diodes whose signal is contaminated by range mixing need to be disregarded. It was hypothesized that analysis of the skewness and kurtosis of the dose rate profile per RMW cycle could be used to determine range mixing.^8^ In this work, measurements and simulations were conducted in homogeneous media in order to establish accurate determination of WEPL through simulated dose rate profiles. Future studies will establish the accuracy of the simulation of detector response in the presence of range mixing by taking advantage of these properties.

The present work focused on implementation of a simulation‐derived calibration fit for detector response for the purposes of range verification in a passively scattered proton beam delivery. This is realized due to the presence of the range modulator wheel which rotates at a fixed rotational speed, and thus dose rate profiles on the scale of the RMW cycle can be correlated to depth in medium. However, the same principles have been investigated for proton radiography in an active scanned beam delivery based on an energy‐resolved dose measurement methodology.^17^, ^18^

## 5 CONCLUSION

A proof of principle was demonstrated for using TOPAS to establish the dose rate profile for a given WEPL to within 3.4 mm in a patient CT and to within 2.4 mm in water‐equivalent plastic. In the region where the detector is expected to lie, within 1 cm of the range of the ‘scout’ beam, this accuracy was shown to be within 1 mm. This enables performing the calibration procedure of the time‐resolved diode dosimetry system without physical measurements. TOPAS simulations of dose rate profiles per RMW cycle established a fit to WEPL with an adjusted R^2^ ≥ 0.999 in water‐equivalent plastic as compared to measurements for three scout SOBPs.

## CONFLICTS OF INTEREST

The authors have no relevant conflicts of interest to disclose.
